# Operations on Typhoid Ulcers

**Published:** 1899-03-04

**Authors:** 


### OPERATIONS ON TYPHOID ULCERS.

We are glad to note that records of successful cases of operations for perforation of the intestine in typhoid fever are coming to hand. Three cases of such treatment, one of which was successful, are reported by Mr. Platt, of Manchester, while in the *St. Thomas's Hospital Gazette* a successful case is also recorded. The latter was a female, aged 11 years, who, after suffering from an attack of enteric fever, had a relapse, in the course of which, *i.e.*, about six weeks from the first onset of the fever, she had a severe attack of abdominal pain and vomiting with a tender and motionless abdomen. The abdomen was opened in the middle line, and a large quantity of sero-purulent fluid with a faint fæcal odour escaped. A perforation was found on the anterior surface of the cæcum, about one-eighth of an inch in diameter, with thick walls, and a considerable amount of surrounding induration. The ulcer was partially excised, and was closed by Lembert sutures. The abdomen was irrigated with sterilised water. The pelvis and the right loin were drained by tubes, and the upper part of the abdomen by gauze. The after-course of the case was somewhat tempestuous. There was much vomiting, the abdomen became distended, the wound gaped and showed no signs of repair, while a large area of gut was visible; a parotid bubo developed, and also an abscess at the site where saline injections had been used, and both knees became the seat of effusion. Finally, the abdomen was re-sutured, the parotid bubo and the abscess were opened, and antistreptococcic serum was systematically used; and, although there was another relapse, the child ultimately did well.

Mr. Platt's successful case was a man aged 37 years, who was admitted suffering from what appeared to be a mild attack of enteric fever. On the day after admission, however, symptoms of perforation occurred, and 22 hours after the probable onset of the perforation the operation was done. Incision four inches long in right semilunar line; gas in peritoneum and a little feculent fluid; great distension; considerable eventration necessary before perforation could be discovered; perforation a quarter of an inch in length, opposite mesenteric attachment, in loop of ileum near pelvic brim; edges turned in and peritoneal surfaces united by continuous silk suture. Free irrigation with hot saline fluid; rubber drainage down into pelvis. The wound gradually closed, but there was considerable distension, and the tongue became dry. In other words the fever ran its course, and, indeed, the patient went through a relapse, but after this he rapidly recovered.

